# Experiences of adults with opioid-treated chronic low back pain during the COVID-19 pandemic: A cross-sectional survey study

**DOI:** 10.1097/MD.0000000000034885

**Published:** 2023-10-13

**Authors:** Aleksandra E. Zgierska, Cindy A. Burzinski, Eric L. Garland, Bruce Barrett, Robert P. Lennon, Roger L. Brown, Anthony R. Schiefelbein, Yoshio Nakamura, Barbara Stahlman, Robert N. Jamison, Robert R. Edwards

**Affiliations:** a Departments of Family and Community Medicine, Public Health Sciences, and Anesthesiology and Perioperative Medicine, Pennsylvania State University College of Medicine, Hershey, PA; b University of Wisconsin-Madison, School of Medicine and Public Health, Department of Family Medicine and Community Health, Madison, WI; c University of Utah, College of Social Work, Salt Lake City, UT; d Department of Family and Community Medicine, and Law School, Pennsylvania State University College of Medicine, Hershey, PA; e University of Wisconsin-Madison, School of Nursing, Madison, WI; f Department of Anesthesiology, Division of Pain Medicine, Pain Research Center, University of Utah School of Medicine, Salt Lake City, UT; g Department of Family and Community Medicine, Pennsylvania State University College of Medicine, Hershey, PA; h Departments of Anesthesiology, Perioperative and Pain Medicine and Psychiatry, Harvard Medical School, Brigham and Women’s Hospital, Chestnut Hill, MA.

**Keywords:** chronic low back pain, coronavirus, COVID-19 pandemic, opioid therapy

## Abstract

This study aimed to evaluate the impact of the COVID-19 pandemic on adults with opioid-treated chronic low back pain (CLBP), an understudied area. Participants in a “parent” clinical trial of non-pharmacologic treatments for CLBP were invited to complete a one-time survey on the perceived pandemic impact across several CLBP- and opioid therapy-related domains. Participant clinical and other characteristics were derived from the parent study’s data. Descriptive statistics and latent class analysis analyzed quantitative data; qualitative thematic analysis was applied to qualitative data. The survey was completed by 480 respondents from June 2020 to August 2021. The majority reported a negative pandemic impact on their life (84.8%), with worsened enjoyment of life (74.6%), mental health (74.4%), pain (53.8%), pain-coping skills (49.7%), and finances (45.3%). One-fifth (19.4%) of respondents noted increased use of prescribed opioids; at the same time, decreased access to medication and overall healthcare was reported by 11.3% and 61.6% of respondents, respectively. Latent class analysis of the COVID-19 survey responses revealed 2 patterns of pandemic-related impact; those with worse pandemic-associated harms (n = 106) had an overall worse health profile compared to those with a lesser pandemic impact. The pandemic substantially affected all domains of relevant health-related outcomes as well as healthcare access, general wellbeing, and financial stability among adults with opioid-treated CLBP. A more nuanced evaluation revealed a heterogeneity of experiences, underscoring the need for both increased overall support for this population and for an individualized approach to mitigate harms induced by pandemic or similar crises.

## 1. Introduction

Since its onset in early 2020, the coronavirus disease-2019 (COVID-19) pandemic has had devastating impacts on the delivery of healthcare services, including the management of opioid-treated chronic pain.^[1]^ Social and physical distancing mandates, strained healthcare systems, and the closures of clinics and other public health services have disrupted continuity of care that is critical for those treated with long-term opioids. This could have contributed to intentional or unintentional accelerated opioid tapering or abrupt discontinuation, with potentially deleterious impacts, such as increased risk of overdose and mental health crisis.^[2]^ The mental health toll of the COVID-19 pandemic, including increased stress, anxiety and depression associated with social isolation and fear of disease, may have exacerbated chronic pain, further complicating its management and compounding the worsened healthcare access issues.^[3]^

Although telehealth adoption dramatically increased during the pandemic as a means to provide a continuum of care, the use of telehealth for chronic pain management has remained limited, a situation which has negatively impacted pain care.^[3–6]^ Access to non-pharmacological treatments (e.g., procedural approaches, physical therapy, or complementary and integrative health therapies such as massage, acupuncture, or chiropractic care) usually requires in-person care delivery.^[6,7]^ Further, because telehealth requires access to the internet or “smartphone,” as well as some technological skills, vulnerable populations, such as the elderly, people with severe mental and other illnesses, those living in poverty or in rural areas, and minorities may have been especially impacted by the disruptions to in-person care. As a result, those with opioid-treated chronic pain have been more likely to experience disruptions to their usual healthcare during the pandemic and, thus, suffer the consequences of inadequate pain management, opioid discontinuation or tapering, and other associated harms.^[4]^

Individuals treated with long-term opioids are a vulnerable population, particularly susceptible to adverse outcomes stemming from inadequate pain care, opioid-related harms, and care disruptions. Even under ideal circumstances, the management of opioid-treated chronic non-cancer pain is complex, individualized, and lacks clear guidance.^[1,8–10]^ Ineffective treatment of chronic pain places a heavy burden on individuals, heath systems, and societies, producing adverse outcomes, such as uncontrolled pain, decreased function and quality of life, psychological stress, worsened mental health, and increased rates of suicide.^[8,11]^ Since peaking approximately a decade ago, the practice of prescribing opioids to treat chronic pain has been fraught with fears of potential misuse by patients, balanced by the need to reduce suffering. Although the exact prevalence of prescription opioid misuse remains unknown, a recent systematic review estimated rates of misuse among those with chronic pain between 21% and 29%.^[11]^ Clearly, opioid prescriptions are part of the current opioid crisis, with its high rates of overdose fatalities, opioid misuse, and opioid use disorder. The problem has been confounded by a lack of high-quality evidence or consistent findings on opioid misuse risk factors, and elusive clinical guidelines for opioid prescribing in chronic noncancer pain.^[12]^

With drug overdose deaths on the rise, reaching 100,306 fatalities, of which 75% involved an opioid in the 12-month period ending in April 2021.^[13]^ Stigma and fear related to opioids have likely only risen, compounding the effects of pandemic-related care disruption. In general, continuity of care, with regular clinic visits focused on patient-centered feedback and shared decision-making, are critical for increasing safety and effectiveness of chronic pain management; disruptions or delays in care can worsen pain and contribute to adverse health outcomes.^[4,8]^ Early in the pandemic worries about access to care and continuation of proper opioid therapy management were common, yet little was known about the extent and effects of these problems. A recent study found that approximately 30% of individuals with chronic pain reported worsened pain severity and pain interference early in the pandemic, and, among those treated with opioids, 40% experienced either access limitations or had concerns about future access to opioid medications, though this specific sample subset was small.^[14]^

There is a scarcity of evidence on the effects of the pandemic specifically among people with opioid-treated chronic pain, despite the evidence that opioid-treated chronic pain increases the risk for severe complications of SARS-CoV-2 infection, including hospitalizations with intensive care and death.^[15]^ The objective of this cross-sectional survey study was to gain better insight into the experiences of people with opioid-treated chronic low back pain (CLBP) in the midst of the COVID-19 pandemic. To accomplish this goal, the participants from an ongoing large randomized controlled trial (RCT) were invited to complete a one-time survey about the pandemic’s impact on their daily life as well as CLBP, opioid therapy, and care/access-related outcomes described in this manuscript.

## 2. Methods

### 2.1. Study design

This survey study followed the STROBE recommendations for the reporting of cross-sectional observational research.^[16]^ The COVID-19 survey study leveraged the ongoing multisite RCT (N = 770), recruiting a convenience sample of survey respondents from among the parent study participants. The Strategies to Assist with Management of Pain study was designed to assess the comparative effectiveness of an eight-week course of mindfulness meditation versus a matched cognitive behavioral therapy-based intervention in adults with opioid-treated CLBP.^[17]^ Trial participants (enrolled between July 2017 and August 2021) were asked to complete a COVID-19 survey. The parent study and the add-on COVID-19 survey study protocols were approved by the Institutional Review Boards (IRBs) governing the study, with the University of Wisconsin-Madison Health Sciences IRB serving as the IRB of record. All parent study participants and survey respondents provided an informed written consent.

### 2.2. Study population

Eligibility for the parent study was determined based on self-report. The participants were English-speaking adults (21 years old or older) diagnosed with CLBP and treated with daily opioids (≥3 months, ≥15 mg/d morphine-equivalent dose), with an average daily pain intensity rating of at least 3 on a 1 to 10 scale (Brief Pain Inventory, BPI^[18]^), and at least moderate CLBP-related disability (≥21 points on the Oswestry Disability Index, ODI^[19,20]^). Individuals were excluded due to previous formal training in, or current practice of one of the study interventions, diagnosis of psychosis or mania, active within the past 12 months; or current pregnancy.

### 2.3. Study settings

Survey respondents were recruited from among the parent study participants who, in turn, were recruited from primary care or pain medicine specialty outpatient settings, and from the community (e.g., via flyers and media advertisement).

### 2.4. Outcome measures

Self-reported outcome measures were collected at baseline (pre-intervention) and again at 3-, 6-, 9- and 12-months post-entry. The measures included: the average pain intensity (BPI^[18]^), physical function (ODI; BPI’s pain interference^[18]^), morphine-equivalent dose of prescribed opioids (Timeline Followback method^[21,22]^), health-related quality of life (12-item Short Form Survey, SF-12, physical, and mental health components^[23]^), negative affect (Hospital Anxiety Depression Scale, HADS^[24]^), opioid misuse risk (Current Opioid Misuse Measure, Current Opioid Misuse Measure^[25]^), pain catastrophizing (Pain Catastrophizing Scale^[26]^), loneliness (3-item UCLA Loneliness Scale^[27]^), feeling loved (Feeling Loved instrument^[28]^), healthcare utilization and level of productivity (HealthCare Utilization and Productivity Scale^[29]^), and baseline demographics.

The COVID-19 Impact Survey (see Survey, Supplemental Digital Content, which contains the COVID-19 Impact Survey, http://links.lww.com/MD/J617) was developed to gain a deeper understanding of the study population’s experience due to the COVID-19 pandemic. The survey was created with expert and stakeholder input, and existing recommendations,^[30]^ with questions designed for comparability with the parent study’s main outcome measures, allowing capture of outcomes important to patients with opioid-treated CLBP. The final survey consisted of 14 questions, with 10 questions using a 7-point Likert scale (worse to better, with a midpoint of no change) to assess participant-perceived change due to COVID-19 in their pain, general activity, enjoyment of life (following the validated Pain, Enjoyment, General Activity survey questions^[31]^), mental health, pain coping, opioid medication use and access, healthcare access, financial situation, ability to meet basic needs, and life overall. In addition, one question asked about the degree of adherence to public health recommendations for preventing COVID-19 (using a 5-point Likert scale, with responses from negative to positive, with a midpoint of no impact, consistent with other Likert scales used to evaluate COVID-19 preventive recommendation adherence^[32]^). One question asked about personal experience with COVID-19 infection, including hospitalization. Two open-ended questions solicited qualitative responses: *“Please share with us how the COVID-19 pandemic has impacted your experience living with chronic back pain” and “What else would you like to share with us about how the COVID-19 pandemic has impacted your life positively and/or negatively overall?.”*

The COVID-19 Impact Survey was administered online through a REDCap database link emailed to participants or, per participant preference, by phone or on-paper (mailed to the participant with an addressed, stamped return envelope). Survey responses collected by phone or on-paper mail were double-entered (to ensure accuracy) into the REDCap database by research coordinators. Surveys administered from June 30, 2020 to October 14, 2020 involved participants who had already exited or were actively participating in the parent study; from Oct 15, 2020 to August 3, 2021 the surveys were administered only to those enrolling in the parent study as a part of their baseline assessment.

### 2.5. Analytical approach

#### 2.5.1. Quantitative data.

Descriptive statistics (frequencies or means ± standard deviation, SD) were used to assess the participants’ demographic and other characteristics. The occurrence of missing data among the COVID-19 survey items was up to 9%. Analysis of these missing data for the COVID-19 survey items and the ancillary variables using Little’s test indicated the assumption of data missing completely at random could be accepted (Chi-square = 60.0, df = 64, *P* = .62). Subsequently, multiple imputation by chained equations was used to impute the missing data prior to the Latent class analysis (LCA).

LCA was performed to identify classes with distinct profiles based on the patterns of responses to the questions about COVID-19 impact. Responses were categorized into improvement, worsening, or no change due to the pandemic. The goal of LCA was to identify and classify homogeneous subcategories known as latent classes from a distribution of responses to an array of heterogeneous indicator variables.^[33]^ Model selection in LCA is based on model fit and determined by comparing the expected frequencies the model predicts to the cross-classification frequencies. Common parsimony indices applied to LCA include Akaike Information Criterion, Bayesian Information Criterion (BIC), and adjusted BIC.^[34]^ The number of classes selected for the model structure were based on the following criteria: interpretability; parsimony; lowest BIC and adjusted (aBIC) scores; lowest Akaike Information Criterion; relative entropy > 0.7; average posterior probability in each class > 0.75 and up to 10% overlap/cross-membership between noncontiguous clusters; ≥2.5% of total count in each class; and no significant improvement as assessed by the likelihood ratio tests.^[35]^ Mplus Version 8.6 was used to model the LCA.^[36]^ Analysis of items used to characterize the LCA classes was done using multiple mean contrast testing, with comparison tests for categorical variables using Pearson’s Chi-squared test. An ancillary analysis was then conducted contrasting various outcome measure relatives to the homogeneous subcategories. The statistical significance level was established at a two-sided α < 0.05; to add correction for multiple testing, False Discovery Rates were also estimated for the between-class comparisons. These analyses were conducted using Stata Version 17.0.^[37]^

#### 2.5.2. Qualitative data.

Qualitative responses were reviewed and organized into categories and main themes, following the thematic qualitative analysis approach.^[38]^

## 3. Results

### 3.1. Survey completion

Initially, the survey was offered to 533 past and current parent study participants; 301 of them completed it. Starting in October 2020, it was offered to 208 individuals enrolling into the parent study, and completed by 179 of them, with 172 completing it within 30 days of their parent study’s baseline questionnaires.

Therefore, the descriptive analyses of COVID-19 survey responses involved a total of 480 participants, and the LCA analyses, which included the parent study’s baseline outcome measures completed within 30 days of the COVID-19 survey, involved 172 respondents. Qualitative responses from 480 respondents were used for qualitative analysis.

### 3.2. Baseline characteristics (N = 480)

#### 3.2.1. Demographics.

The majority of 480 survey respondents were female (60.6%), Caucasian (83.1%), and non-Hispanic/Latino (87.9%; Table 1). Fourteen percent (14.4%) reported graduating from high school or having a GED, 67.8% reported having some college or a bachelor’s degree, and 16.9% reported having a graduate degree. On average, the respondents were 58.7 ± 10.5 years old, with only 19.5% identifying as currently working, and 43.3% reporting a disability status, primarily due to back pain.

**Table 1:** Demographic, chronic low back pain-related, and clinical characteristics of the study sample (N = 480).

Variable*	Sample (N = 480)
Demographic characteristics	
Gender, n (%)	
Female	291 (60.6)
Male	188 (39.0)
Age, years, mean (SD)	58.7 (10.5)
Ethnicity, n (%)	
Hispanic or Latino	15 (3.1)
Not Hispanic or Latino	422 (87.9)
Race, n (%)	
Black/African American	39 (8.1)
White	399 (83.1)
Other	21 (4.4)
Education, n (%)	
No high school diploma	10 (2.1)
High school graduate or GED	59 (12.3)
Some college, no degree	102 (21.2)
Occupational/technical/vocational program	37 (7.7)
Associate degree: academic program	73 (15.2)
Bachelor’s degree	114 (23.7)
Master’s degree	61 (12.7)
Doctoral degree	20 (4.2)
Employment, n (%)	
Working now	94 (19.5)
Unemployed	29 (6.0)
Disabled	208 (43.3)
Disabled due to back pain	147 (30.6)
Retired	109 (22.7)
Other (includes Other, Sick or maternity leave, Student, Keeping house)	38 (7.9)
Back pain history and perception characteristics (NIH MD)	
CLBP duration, n (%)	
3–6 mo	3 (0.6)
6 mo–1 yr	6 (1.3)
1–5 yr	73 (15.2)
> 5 yr	398 (82.9)
Prior treatments for back pain, Yes, n (%)	
Injections *(e.g., epidural steroid, facet*)	395 (82.3)
Exercise therapy (physical therapy)	447 (93.1)
Psychological counseling	171 (35.6)
History of a low back operation, n (%)	
Yes	214 (44.6)
One operation	93 (19.4)
More than one operation	121 (25.2)
No	263 (55.09)
I feel that my back pain is terrible and it’s never going to get any better…, n (%)	
Agree	231 (48.1)
Disagree	236 (49.1)
It’s not really safe for a person with my back problem to be physically active…, n (%)	
Agree	146 (30.4)
Disagree	326 (67.9)
Clinical characteristics	
Average pain severity (BPI, 0-10 score), mean (SD)	5.9 (1.5)
Function	
Pain interference (BPI, 0-10 score range), mean (SD)	6.4 (1.8)
Back pain-related disability (ODI, 0-100 score range), mean (SD)	47.4 (13.6)
Health-related quality of life (SF-12)	
Physical health composite score (0-100 range), mean (SD)	28.6 (8.2)
Mental health composite score (0-100 range), mean (SD)	42.8 (11.6)
Daily dose of prescribed opioids, past 14 days (TLFB, morphine-equivalent mg/d)	
Average daily dose, mean (SD)	137.3 (520.0)
Average daily dose categories, n (%)	
0 (no opioids)	3 (0.6)
0.1–50 morphine-equivalent mg/d	279 (58.1)
50–89 morphine-equivalent mg/d	64 (13.3)
90 + morphine-equivalent mg/d	91 (18.9)
200 + morphine-equivalent mg/d	43 (8.9)
Opioid misuse risk (COMM)	
Total score (0–68 range), mean (SD)	9.6 (6.9)
Positive screen (score 9+), n (%)	226 (47.0)
Cigarette Smoking Status, n (%)	
Never smoked	194 (40.4)
Used to smoke	191 (39.7)
Current smoker	91 (18.9)
Sleep quality (NIH MD), total score (0-20 range), mean (SD)	10.3 (4.0)
Negative affect (HADS)	
Negative affect, total score (0–42 range), mean (SD)	16.3 (7.5)
Anxiety subscale score (0–21 range), mean (SD)	8.1 (4.4)
Depression subscale score (0–21 range), mean (SD)	8.2 (4.0)
Pain catastrophizing (PCS), total score (0–52 range), mean (SD)	20.2 (11.6)
Loneliness (UCLA LS)	
Total score (3–9 range), mean (SD)	5.8 (2.1)
Positive screen (6 + points), n (%)	270 (56.6)
Feeling loved (FLI)	
Feeling loved by others (0–100 score range), mean (SD)	77.7 (25.7)
Loving yourself (0–100 score range), mean (SD)	68.9 (28.0)
Healthcare utilization and productivity characteristics (HCUPS), past 6 months	
Hospitalized, Yes, # (%)	84 (17.5)
Number of nights hospitalized (n = 84), mean (SD)	7.8 (13.3)
ED visit, Yes, # (%)	150 (31.3)
Number of ED visits (n = 150), mean (SD)	2.5 (2.8)
Clinic visit (“regular” care, urgent care), Yes, # (%)	404 (84.2)
Number of clinic visits (n = 404), mean (SD)	9.0 (30.0)
Mental health care visit, Yes, # (%)	170 (35.4)
Number of mental health care visits (n = 170), mean (SD)	8.7 (9.2)
Addiction care visit, Yes, # (%)	17 (3.5)
Number of addiction care visits (n = 17), number, mean (SD)	8.3 (13.0)
Chores (school/house work) missed, Yes, # (%)	305 (63.5)
Number of whole days of chores missed (n = 305), mean (SD)	41.7 (48.8)
Whole workdays missed (among those “working now,” n = 93), Yes, # (%)	
Whole workdays missed, Yes, # (%)	58 (62.4)
Number of whole workdays missed (n = 58), mean (SD)	10.6 (26.5)

BPI = Brief Pain Inventory, CLBP = Chronic Low Back Pain, COMM = Current Opioid Misuse Measure, FLI = Feeling Loved Instrument, HADS = Hospital Anxiety Depression Scale, HCUPS = HealthCare Utilization and Productivity Survey, NIH MD = National Institutes of Health Minimal Data Set, PCS = Pain Catastrophizing Scale, SF-12 = 12-Item Short Form Health Survey, TLFB = Timeline Followback, UCLA LS = UCLA Loneliness Scale.

* The frequencies/percentages in a given variable category may not total the sample size of 480 or 100% responses as some participants’ responses were unavailable (e.g., missing or marked as declined, unknown or unspecified).

#### 3.2.2. Back pain history and perceptions.

All participants had CLBP at enrollment. Most had tried multiple treatments for their pain including injections (82.3%) and/or physical therapy (93.1%). Close to one-half of the respondents reported at least one prior back surgery (44.6%), and about half identified their back pain as “terrible and never going to get any better” (48.1%). Almost one-third (30.4%) felt their back pain made it unsafe for them to be physically active.

#### 3.2.3. Clinical characteristics.

This sample reported moderately-high pain severity on a daily basis (5.9 ± 1.5) and considerable pain interference with activities of daily living (6.4 ± 1.8). Their average ODI total score (47.4 ± 13.6) implied moderate back pain-related disability and interference with all aspects of life. The SF-12 mental and physical health component scores (42.8 ± 11.6 and 28.6 ± 8.2, respectively) indicated a compromised health-related quality of life, especially physical health. These results paralleled the elevated average scores of negative affect (HADS: 16.3 ± 7.5) and pain catastrophizing (20.2 ± 11.6), and subpar sleep quality (10.3 ± 4.0). The survey respondents were treated, on average, with a high daily dose of prescribed opioids (137.3 ± 520.0 morphine-equivalent mg/day). However, a wide variability was noted, with 3 individuals having discontinued taking opioids. Approximately one-half (47.0%) screened positive for aberrant opioid use behaviors based on their COMM score ≥ 9 points. Close to one-fifth of the sample (18.9%) reported currently smoking tobacco. They overall reported increased loneliness and/or social isolation, with 56.6% screening positive for loneliness. On a 0-100 visual analog scale, they averaged 77.7 (SD 25.7) for how much they felt loved, and 68.9 (SD 28.0) for how much they loved themselves.

#### 3.2.4. Healthcare utilization and productivity.

The survey respondents reported a heavy healthcare resource utilization at baseline, with close to one-fifth experiencing hospitalization and one-third reporting an emergency department visit within the past 6 months. The majority (84.2%) were seen at a clinic for routine or urgent care, 35.4% reported having an outpatient mental health care and 3.5% reported having an outpatient addiction care visit. Two-thirds (63.5%) reported missing entire days of housework or school responsibilities, and, among those who reported working, 62.4% missed at least 1 day of work due to the pain.

### 3.3. COVID-19 pandemic impact (N = 480)

#### 3.3.1. Chronic pain-relevant outcomes.

A large proportion of the respondents reported experiencing negative impact of the COVID-19 pandemic on their life and across all domains (84.8%), with worsened enjoyment of life (74.6% of the respondents), mental health (74.4%), general activity (68.1%), pain (53.8%), and the ability to cope with pain (49.7%) in the past month (Table 2). One-fifth (19.4%) of the respondents reported increased use of prescribed opioids due to the pandemic. At the same time, access to prescription opioids decreased for 11.3% of respondents and the overall access to healthcare, including regular healthcare provider visits and specialty treatments, decreased for 61.6% of all respondents, with close to one-half noting a worse financial situation (45.3%) or worsened ability to meet basic needs (42.6%). Only a small proportion of the respondents reported improvements in chronic pain-relevant outcomes, with improvement in general activity (8.1%), healthcare access (6.1%), finances (6.9%) or life overall (3.3%). While 3.7% reported a decreased use of opioid medication, it was unclear if this was because of their provider or because of their own decision.

**Table 2:** Impact of the COVID-19 pandemic on chronic pain-relevant outcomes and behaviors aimed at reducing the spread of SARS-CoV-2 virus (N = 480).

	n (%)	Change categories	Percent of change
Pandemic impact on chronic pain-relevant outcomes			
Due to the pandemic, pain on average (past month)			
Very much increased	48 (10.0)	Worse	53.8
Much increased	86 (17.9)		
Minimally increased	124 (25.8)		
Not changed	198 (41.3)	No change	41.3
Minimally decreased	16 (3.3)	Improved	5.0
Much decreased	5 (1.0)		
Very much decreased	3 (0.6)		
Due to the pandemic, general activity (past month)			
Very much worse	68 (14.2)	Worse	68.1
Much worse	138 (28.8)		
Minimally worse	120 (25.1)		
Not changed	114 (23.8)	No change	23.8
Minimally improved	26 (5.4)	Improved	8.1
Much improved	10 (2.1)		
Very much improved	3 (0.6)		
Due to the pandemic, enjoyment of life (past month)			
Very much worse	61 (12.8)	Worse	74.6
Much worse	130 (27.3)		
Minimally worse	165 (34.6)		
Not changed	92 (19.3)	No change	19.3
Minimally improved	21 (4.4)	Improved	6.1
Much improved	6 (1.3)		
Very much improved	2 (0.4)		
Due to the pandemic, ability to cope with pain (past month)			
Very much worse	25 (5.2)	Worse	49.7
Much worse	80 (16.7)		
Minimally worse	133 (27.8)		
Not changed	215 (44.9)	No change	44.7
Minimally improved	17 (3.6)	Improved	5.4
Much improved	7 (1.5)		
Very much improved	2 (0.4)		
Due to the pandemic, mental health, including stress, anxiety or depression symptoms (past month)			
Very much worse	64 (13.4)	Worse	74.4
Much worse	96 (20.0)		
Minimally worse	197 (41.1)		
Not changed	101 (21.1)	No change	21.1
Minimally improved	13 (2.7)	Improved	4.4
Much improved	6 (1.3)		
Very much improved	2 (0.4)		
Due to the pandemic, use of prescribed opioid medications (past month)*			
Very much increased	6 (1.4)	Increased	19.4
Much increased	20 (4.6)		
Minimally increased	58 (13.4)		
Not changed	333 (76.9)	Neutral	76.9
Minimally decreased	11 (2.5)	Decreased	3.7
Much decreased	3 (0.7)		
Very much decreased	2 (0.5)		
Due to the pandemic, access to prescribed opioid medications (past month)*			
Very much decreased	4 (0.9)	Worse	11.3
Much decreased	7 (1.6)		
Minimally decreased	38 (8.8)		
Not changed	362 (83.4)	Neutral	83.4
Minimally increased	10 (2.3)	Improved	5.3
Much increased	10 (2.3)		
Very much increased	3 (0.7)		
Due to the pandemic, access to healthcare (including regular healthcare provider and specialty treatments)			
Very much worse	45 (9.4)	Worse	61.6
Much worse	96 (20.1)		
Minimally worse	153 (32.0)		
Not changed	155 (32.4)	No change	32.3
Minimally improved	17 (3.6)	Improved	6.1
Much improved	10 (2.1)		
Very much improved	2 (0.4)		
Due to the pandemic, financial situation (including income, savings, ability to pay bills, etc.)			
Very much worse	49 (10.3)	Worse	45.3
Much worse	65 (13.7)		
Minimally worse	104 (21.9)		
Not changed	225 (47.3)	No change	47.0
Minimally improved	25 (5.3)	Improved	6.9
Much improved	2 (0.4)		
Very much improved	6 (1.3)		
Due to the pandemic, ability to meet basic needs (including housing, food, essential supplies, etc.)			
Very much worse	21 (4.4)	Worse	42.6
Much worse	52 (10.9)		
Minimally worse	132 (27.6)		
Not changed	255 (53.2)	No change	53.2
Minimally improved	14 (2.9)	Improved	4.0
Much improved	3 (0.6)		
Very much improved	2 (0.4)		
Due to the pandemic, life overall			
Very negatively overall	113 (23.8)	Worse	84.8
Somewhat negatively overall	163 (34.3)		
A little bit negatively overall	131 (27.6)		
Not impacted overall	52 (11.0)	No change	10.9
A little bit positively overall	8 (1.7)	Improved	3.3
Somewhat positively overall	6 (1.3)		
Very positively overall	2 (0.4)		
Pandemic impact on practice of public health recommendations to reduce the spread of SARS-CoV-2 virus			
Wash hands often with soap and water for at least 20 seconds, especially after being in a public place, or after blowing nose, coughing, or sneezing			
Never	1 (0.2)	Never-rarely	0.4
Rarely	1 (0.2)		
Some of the time	28 (5.9)	Some of the time	5.9
Most of the time	117 (24.5)	Most or all the time	93.7
All of the time	330 (69.2)		
Wear a cloth face cover (facemask) when out in public			
Never	4 (0.9)	Never-rarely	2.8
Rarely	9 (1.9)		
Some of the time	25 (5.3)	Some of the time	8.1
Most of the time	71 (15.2)	Most or all the time	91.9
All of the time	359 (76.7)		
Avoid touching eyes, nose, and mouth with unwashed hands			
Never	5 (1.1)	Never-rarely	5.1
Rarely	19 (4.1)		
Some of the time	62 (13.2)	Some of the time	13.1
Most of the time	182 (38.8)	Most or all the time	81.7
All of the time	201 (42.9)		
Cover mouth and nose with a tissue when coughing or sneezing or use the inside of the elbow			
Never	0 (0.0)	Never-rarely	1.5
Rarely	7 (1.5)		
Some of the time	12 (2.6)	Some of the time	2.6
Most of the time	70 (15.2)	Most or all the time	95.9
All of the time	373 (80.7)		
Stay at home when feeling unwell			
Never	0 (0.0)	Never-rarely	0.2
Rarely	1 (0.2)		
Some of the time	12 (2.7)	Some of the time	2.7
Most of the time	54 (12.2)	Most or all the time	97.1
All of the time	377 (84.9)		
Stay at least 6 feet (about 2 arms’ length) from other people when out of home			
Never	1 (0.2)	Never-rarely	0.8
Rarely	3 (0.6)		
Some of the time	28 (6.0)	Some of the time	6.0
Most of the time	134 (28.6)	Most or all the time	93.2
All of the time	303 (64.6)		
Stay out of crowded places and avoid mass gathering			
Never	0 (0.0)	Never-rarely	0.8
Rarely	4 (0.9)		
Some of the time	19 (4.1)	Some of the time	4.1
Most of the time	82 (17.7)	Most or all the time	95.0
All of the time	358 (77.3)		

* Percentages calculated for 435 people (90.6% of the sample) who reported current treatment with opioid medications; of note, 42 people responded they were not currently treated with opioids, and 3 people did not provide an answer to this question.

#### 3.3.2. Practices to reduce the viral spread.

The respondents reported high adherence to public health-recommended preventive practices (Table 2), with over 90% endorsing hand washing, wearing a face mask when in public, covering mouth/nose when coughing or sneezing, staying at home when not feeling well, avoiding gatherings or crowded places, and maintaining physical distancing outside of their homes most or all of the time. Only 7 respondents (1.5%) reported being hospitalized due to COVID-19.

#### 3.3.3. Qualitative findings.

When asked to describe in their own words the pandemic’s impact on their experience living with CLBP and life in general, participant responses formed 3 major categories: positive impact or no impact (a small proportion of respondents), or negative impact (the majority of provided comments).

The perceived positive effects of the pandemic coalesced around several themes. Some participants noted increased closeness with friends and family members (sometimes despite physical distancing), and better ability to identify and focus on what is important in life, fostered by fewer distractions and more time spent on self-care. A few participants mentioned improved CLBP-related symptoms (e.g., lessened pain or increased functional capacity, typically linking it to exercising more and taking better care of oneself), accompanied by the ability to reduce opioid use due to positive changes. Several participants described spontaneous acts of kindness and support they observed or experienced brought on by the pandemic as uplifting and bringing hope.

The descriptions of “no change” were typically very brief, often consisting of just a few words. Some explained that they would not have expected any pandemic impact on their CLBP because these were unrelated conditions/concepts. Among these participants, lack of perceived substantial pandemic harms on life overall was often noted.

The majority described a harrowing picture of the pandemic’s impact on themselves and their families (Table 3). Many reported worsened pain and function, linking it to several aspects of the pandemic-related restrictions and stressors. Reduced healthcare/ medication access was accompanied by anxiety, stress, and fear related to both the potentially life-threatening consequences of COVID-19 for themselves and their families, and by social and physical isolation, loneliness, and financial problems. All these stressors contributed to worse mental health and sleep challenges, which in turn made CLBP worse. Some participants described how lack of external help (from family, friends, or hired helpers) they had received prior to the pandemic or pandemic-caused financial instability forced them to take up chores they had done previously, and now these responsibilities (e.g., house cleaning, working more to financially support the family) caused more pain, along with other deleterious consequences (e.g., worse mental health). For some participants the social isolation and staying at home made them feel trapped and more depressed, and substantially restricted their physical activity. Altogether, this led some individuals to increase their dose of opioids to better manage pain, while others described struggles with being able to access prescribed opioids as a major stressor. It was not uncommon for participants to describe their disappointment about and frustration with what they viewed as a politicization of pandemic and public health preventive practices, while, at the same time, many described strict adherence to these practices due to the fear of contracting COVID-19, which they viewed as dangerous for people with complex health problems, such as themselves.

**Table 3:** Experiences of adults (n = 480) with opioid-treated chronic low back pain during the COVID-19 pandemic: findings from the thematic qualitative analysis of comments about the pandemic’s impact on living with chronic pain and life overall.

Themes	Representative quotes
Category: Positive impact of the pandemic	
*Increased closeness with friends or family*	“Because my daughter has been home with her [children], she has made the effort to FaceTime with me almost every day and also send videos and pictures, plus I usually get to read a naptime story to the 3 year old. That’s much more accessibility than I had when she was working […]. I’ve had something to look forward to every day while I’ve been isolating.” “I have developed closer relationships with my family. For example I now regularly speak to my brother […]. With my other sibs we speak and video party/game night at regular intervals which never happened in the past.” “on the positive side, it’s helped me to evaluate things more compassionately and with more patience for others with the mental health challenges they face. […] I think those skills […] has helped take inventory of what’s going on, instead of being introverted with pain, developing the mindset and automatic response to any kind of difficult stimulation (mental or physicals) I was able to change and look outwardly to apply those skills […].” “I’ve made new friends on Zoom.” “I have been able to participate in more programs via zoom or other virtual meeting venues.”
*Increased focus on what really matters and on self-care*	“[…] it has made myself and all I love to re-prioritize. To remember what truly is the most important thing...Love.” “It did help me slow down a bit which did give me more determination to find tools to help me with my pain. It also helped me to pin point more things of what was working and not working and ended the loop of trying to seek tons of different solutions. The running from solution to solution stopped and I found more tools that I could do myself.” “Since the gardening season has started, I’ve been focused on getting out to purchase seeds, plants and soil and then prepping and planting my garden and flowers. This [… has given] me a sense of joy and accomplishment.” “The COVID pandemic had redirected my life in positive ways. I now know that preparedness ahead of time is everything. […] Having my pills on time, setting my next appointment with my pain manager etc. have taken precedence over other things.”
*Improved CLBP-related symptoms or care*	“I will say that I have been working to manage my stress level […]. My use of anxiety meds and sleeping meds has actually gone down due to this management.” “Thankful for having a smart Doctor who didn’t allow it to interrupt my Prescription refills.” “[gardening] has caused some increase in pain and related increase in use of narcotics, but has also helped to give me a sense of joy and accomplishment as I look at my garden and flowers.” “Telemedicine is perfect for me, since driving hurts my back and right knee.” “you learn that you can do a lot of things virtually and still be okay. in a way life has been a little more relaxing.”
*Improved outlook on humanity*	“The positive things people look out for you, check on you (do you need anything). […] overall I thank them very much for their help.” “Most of my answers to this survey have not been positive, but I have seen extraordinary acts of kindness, almost random, in the sense that people who are asking via the internet offering services or offering does anybody need something, things like that, people rising to the occasion of how they can help people.”
Category: No impact of the pandemic	
*No change*	“zero impact...” “Has been about the same. Still have back pain every day.”
*Not linking change to the pandemic*	“The back and hip pain have been significantly worse; however, I can’t relate it to COVID-19.” “COVID-19 has absolutely nothing to do with chronic back pain. They are mutually exclusive diagnosis’s.”
Category: Negative impact of the pandemic	
*Worsened pain, function, quality of life*	“NONE POSITIVELY ALL NEGATIVELY “ “probably like a lot of other people that deal with chronic pain it just made a bad situation worse.” “I believe my future is grim.” “I am very limited in what I can physically do and COVID-19 made me very aware of how little I can accomplish. Since I spent all of my days shut-in it focused me more on my pain. Every little bit of it.“ “Because of having nothing to do at all, I have had time to think about my pain more [with worsened pain].” “Not much to do and very little social contact. Seems like the pain is the only thing that is permanent and sometimes my only focus, seems worse at times.” “It impacts every part of my life and it has increased my pain.” “It does have an effect on people with chronic back pain. I try to keep my mind active in doing things, but everything has stopped, so I’m left to just deal with the pain. I have to ‘feel’ the pain more than what I normally would, like getting up and going places or doing something.” “Negatively. I hope it will be over soon, but otherwise, it has affected my financial situation, my mental and physical health, and it has become bad because of it.” “My joints and lower back pain hurt worse when I had covid and had stayed with me since.”
*Worse pain, anxiety, stress due to the pandemic-related restrictions and financial problems.*	“Omgosh it’s ruined my/our life. I fear losing home, auto, my meds are so expensive and moneys tight. We live pay Check to pay check and I have no life now. […] I’ve been house bound for 10 months. I’m so isolated plus my auto broke down so I can’t Even go for a ride.” “The pandemic has increased the feelings of isolation and increased depression due to the lack of personal interaction and restricted abilities to get out of the house.” “Sheltering at home without normal activity or interaction caused me to focus more on my pain. Boredom and not working has been a mental & emotional burden. Money worries creates anxiety which increases pain.” “I can’t think of anything positive at the moment. I didn’t think it would change financially, but my financial needs are greater. I would most likely think that being at home more or not being able to get out it wouldn’t change, we’re on a fixed income but we’re using more money now for safer transportation or paying someone to do the shopping for us, just getting regular household needs. Before then I had no problem doing the shopping or doing the cooking, but it’s gotten a lot worse since the pandemic.” “Finances are stretched which stresses me and so I hurt from stress and anxiety. I then don’t sleep and so I hurt more because I toss and turn. It affects those with chronic pain a lot more because there are situations we can’t help. So we stress and stressing hurts. I work on things I was taught but things don’t always work out.” “[…] Not being to physically touch one another. Having to try and go to all of my doctor appointments and hoping I can have one of my kids with me to help me walk, and remember all the info the Dr is giving me. And my L3 disc getting worse and worse […].” “Personally, most of the increased pain was due, in part, to increased financial stress which ‘blocked’ my ability to ‘keep the pain quiet’ via my mind and breathing techniques.” “Well, I am unable to walk more than a block due to my hip pain, so my ability to get outside and get fresh air has been severely hampered. I can’t even walk my dog the way she needs to be walked. And the pain is so exhausting, so I am even unable to participate in Zoom meetings with my friends and family. I am simply too tired by the end of the day. In short, it has sucked.” “I have had to cook more often with restaurants being closed. More cooking meant more grocery shopping and more dishes to clean. Had to stop having a cleaning lady to avoid getting Covid. Trying to clean my house has been practically impossible for me to do a thorough job.” “Not having ready access to my medical providers and the isolation of sheltering in place to reduce exposure to COVID-19 has taken quite a toll on me emotionally. This in turn has increased my sensitivity to my chronic pain issues. I have experienced increased levels of pain, depression, and lack of sleep.” “It’s a little worse. The isolation made me so that I didn’t have any distractions. The isolation made me more aware, and the depression made it worse. Seems like when I get depressed everything hurts more. […] I was depressed because I couldn’t see my […] children.” “I was close to getting housing and now because of COVID that’s gone. It’s impacted all areas of my life, literally. Socially, emotionally, I mean everything. It screwed up everything.” “Having all the schools about down increased pain by causing more stress and work with kids at home.”
*Stressors made mental health, sleep, and, in turn, pain worse.*	“The biggest impact of the pandemic has been on my mental health. Losing all of my activities outside the home caused me to go into depression, plus the anxiety over the potential for getting sick. […] Mentally I am not yet back to where I was pre-pandemic.” “I have experienced much anxiety and my depression has gotten much worse making it harder to deal with my pain.” “Already very isolated and in pain, gross increase in those and terrible chaos in everyday life has increased the suicidal ideations I’ve had since childhood EXPONENTIALLY. It’s good to have an increase to 2 mental health appointments per week virtually but everything is just always Terrible- and then.... PANDEMIC.”
*Social distancing caused isolation and restricted physical activity, with negative impact*s	“I hate it, I hate it, I hate it. I miss my family, I miss people, I miss swimming and all these things cause me to be depressed almost always. Also my weight gain caused by inactivity is extremely depressing as well and causes more pain because my back is having to support more.” “Felt even more isolated. My husband went to work and my daughter to school but I had to stay home to avoid getting Covid. My life felt like the movie Groundhog Day-just kept repeating day after day.” “Not going anywhere and just laying around sucks. I feel like dying. I am a prisoner in my own home.” “I ‘Stayed home, stayed safe’ and ended up with a foot long blood clot in the artery in my left leg.” “During the shutdown period when all of my usual activities were canceled, I had to manage my back pain on my own (i.e.. find substitutes for social activities that may have helped manage the pain). Also was much more aware of my pain since I did not have the distraction of social activities.” “Although I continue to exercise and do stretch classes on line, I find that my activity level has diminished. I do not go out as often as I did before the pandemic and I don’t walk as much.” “It’s more difficult finding comfortable ways to meet socially outside and 6’ apart. I can’t sit on picnic benches, metal chairs, on the ground, etc. I must always bring my own chair, which altho it’s portable, it’s a bit too heavy for me to carry.”
*Reduced healthcare or medication access*	“The most significant impact was the loss of access to health care providers, hospital, clinic, doctors, physical therapists, massage therapists and warm water therapy pool. All of those have resulted in a lack of care, exercise and general decline in my physical status which results in increased pain.” “Once my pain clinic […], physical therapy […] and private pilates sessions were shut down, I was basically left with no medical support for my pain for more than 2 months. […] I truly understand that the situation is unprecedented, and healthcare providers have no roadmap for how to proceed, but it has been a miserable, frightening experience from a patient standpoint. I am frankly terrified that I will have to live without support again. It is difficult to express just how awful it was to have no one to help me during this time.” “Healthcare providers are overwhelmed by covid. They are burned out, unhappy and my needs are not important.” “Not wanting to bother my health team knowing they are being over whelm with Covid.” “It is hard to get my medications; supply and getting prescriptions renewed.” “I was unable to be seen by my Pain Management clinic where I was being treated with […] injections for my back pain. Without these injections, my life went back to a barely functional existence. I was unable to sleep in a bed and was barely able to sleep in my recliner, I was barely able to do the grocery shopping and house cleaning let alone take care of myself […].” “It took my doctor out of my help, no more prescription.”
*Opioid medication dose/ access issues*	“My new Dr took away ALL my opioids & w/the pandemic it seems THAT aspect of my life is no longer a concern. Even getting otc pain medication is a feat.” “Could not have my biweekly therapeutic massage so had to have opioids increased so I could sleep. Pain keeping me up.” “The higher level of stress and back pain and my anxiety and feelings of helplessness led me to relapse and take opioids again. It did help with the pain though.” “Well, not having access to the hot tub and not having a way to get that thermal therapy i depend on has been frustrating having to mitigate that discomfort with other modalities and I had to take more opioids to compensate.” “In the middle of this pandemic, opioid supplies were scarce. We had very long waiting lines at our pharmacy. So, I did have to wait sometimes for my opioid prescriptions more than a day; so I had to make sure that my pain provider would call them in or hand me the prescription before it was due... [sometimes] I had to wait for the shipment to make it to our pharmacy.”
*Fear of COVID-19 infection and serious complications*	“I wait until i can’t stand the pain before I go to the chiropractor, i don’t go to my regular doctor because one of the nurses there told me she and others had been exposed and where still working (when I went in for my blood tests).” “Since my health is so very complex, my daughter had to stay home from school. So she could not infect me with the virus.” “i also have extreme anxiety and PTSD attacks from wearing a mask and hearing only bad reports from the media.” “The virtual doctor appointments are not ideal, on the other hand PT makes me feel unsafe.” “Don’t want to go out so not to get covid.” “I am at high risk. Since life has gotten somewhat back to normal, I am not so depressed, but there is still the anxiety over the covid variants, people not wanting to get vaccinated, etc.” “The level of stress it has caused has negatively impacted me. […] Daily dwelling on errands about where to go and how busy will the grocery stores or banks be. There is nothing we can do right now that will not have a COVID thought attached to it. Mentally exhausted then increases pain. COVID has made everything more intensified.” “Negatively, I have become dependent on my children to provide me with food and medical supplies. We are in agreement that we should all self isolate in order to stay healthy. Altho I do see friends in an open air atmosphere, it is not the same. My ‘retail therapy’ (including shopping etc.) no longer exists.” “I wasn’t able to leave my home and I didn’t. That is why I never contact the virus.” “Just in not getting out as much. I talk to my daughter but rarely see her. I do feel sort of stuck at home because I don’t want to get sick and I worry about it.” “It’s just hard all the way around. Because some things I just can’t do anymore. And I’m wearing a mask all the time and washing my hands all the time. It’s getting to be hectic for me, I don’t know about nobody else.” “It makes me feel even more isolated than I kind of already did when I try to think of things that I would want to do physically, the virus is stopping me from doing them.” “I can’t get up as much as I would like to. I would like to try some exercises and things like that and I can’t get to the gym because it’s not safe. Going out to visit family and friends, I cannot do because it’s not safe.”
*Frustration with handling of the pandemic and preventive practices*	“I have seen issues that I see as common sense safety issues like masks become political hand grenades and was astonished.” “Social media has become distressing, the news has been overwhelming.” “What negatively impacted my life was the purposeful political response to the virus.” “[…] personally I think the media is blowing it all out of proportion I think we should focus on what we can do individually to control it. I don’t see people doing what they can to mitigate the risk. People don’t want to do what they’re asked to do and that’s why we are seeing an increase and it’s very frustrating for me because if they did, we probably wouldn’t see the increases we do and have the issues with it. […] progress seems slow and the politics are intense.” “Listening to the news, social media especially about the politicization of the Covid has been depressing.” “I would say my level of anxiety around the pandemic and adjacent political environment was pretty extreme at the start of things. I’ve been able to work through a lot of this, but I still harbor a decent amount of anxiety around the state of things.” “Has increased anxiety and anger over political atmosphere.” “Seems like it is almost a political statement whether a person wears a mask or not.”

### 3.4. COVID-19 pandemic impact (n = 172): latent class analysis (LCA)

The COVID-19 survey was completed by 172 participants in conjunction with their parent study’s baseline assessment, enabling the analyses of COVID-19-specific responses in relation to the broader set of outcomes collected for the main study, and were included in the LCA.

The LCA model identified 2 distinct classes of participants based on their responses about the COVID-19 pandemic impact (Class 1: n = 66; Class 2: n = 106) that showed better fit than other multi-class models (*P* < .001). Average posterior probabilities were high for Class 1 (0.963) and Class 2 (0.961), with only a 0.58% overlap in the model, well below the accepted 10% benchmark, with both classes maintaining total counts above 2.5% of the sample (with Class 1 at 39.1%, and Class 2 at 60.9%).

As presented in Table 4, compared to Class 1 (n = 66), Class 2 (n = 106) participants reported worse pandemic-associated harms across all COVID-19 survey questions, including worsened average pain (59.4% vs 20.0%, respectively), general activity (81.9% vs 21.2%, respectively), enjoyment of life (95.2% vs 18.1%, respectively), ability to cope with pain (62.3% vs 3.0%, respectively), and mental health symptoms (95.2% vs 24.2%). In addition, 97.1% of Class 2 noted negative pandemic impact on life overall, compared to 72.3% Class 1 participants. Approximately twice as many Class 2 participants, compared to Class 1, reported a worse financial situation (51.4% vs 30.3%, respectively), and a worse ability to meet basic needs (49.5% vs 18.1%, respectively). Class 2 participants were also more likely than Class 1 to report increased opioid use (18.1% vs 1.6%, respectively) as well as challenges with access to both opioid medication (decreased: 13.3% vs 3.1%, respectively) and general healthcare (decreased: 60.6% vs 28.7%, respectively).

**Table 4:** Latent Class Analysis of the COVID-19 Impact Survey responses: Class 2 participants (n = 106) more frequently reported harms due to the COVID-19 pandemic compared to Class 1 participants (n = 66).

Question about the COVID-19 pandemic’s impact	Pandemic impact	Class 1 n (%)	Class 2 n (%)	*P* value	FDR†
Due to the pandemic, pain on average (past month)	Worse	13 (20.0)	63 (59.4)	<.001	0.005
	No change	48 (73.9)	40 (37.7)		
	Improved	4 (6.2)	3 (2.8)		
Due to the pandemic, general activity (past month)	Worse	14 (21.2)	86 (81.9)	<.001	0.005
	No change	38 (57.5)	14 (13.3)		
	Improved	14 (21.2)	5 (4.8)		
Due to the pandemic, enjoyment of life (past month)	Worse	12 (18.1)	100 (95.2)	<.001	0.005
	No change	43 (65.1)	3 (2.9)		
	Improved	11 (16.7)	2 (1.9)		
Due to the pandemic, ability to cope with pain (past month)	Worse	2 (3.0)	66 (62.3)	<.001	0.005
	No change	57 (86.3)	39 (36.8)		
	Improved	7 (10.6)	1 (0.9)		
Due to the pandemic, mental health, including stress, anxiety or depression symptoms (past month)	Worse	16 (24.2)	100 (95.2)	<.001	0.005
	No change	38 (57.5)	5 (4.8)		
	Improved	12 (18.1)	0 (0.0)		
Due to the pandemic, use of prescribed opioid medications (past month)*	Increased	1 (1.6)	19 (18.1)	.003	0.013
	No change	62 (96.8)	82 (78.1)		
	Decreased	1 (1.6)	4 (3.8)		
Due to the pandemic, access to prescribed opioid medications (past month)*	Decreased	2 (3.1)	14 (13.3)	.022	0.067
	No change	61 (95.3)	84 (80.0)		
	Increased	1 (1.6)	7 (6.7)		
Due to the pandemic, access to healthcare (including regular healthcare provider and specialty treatments)	Worse	19 (28.7)	63 (60.6)	.0002	0.005
	No change	40 (60.6)	34 (32.7)		
	Improved	7 (10.6)	7 (6.7)		
Due to the pandemic, financial situation (including income, savings, ability to pay bills, etc.)	Worse	20 (30.3)	54 (51.4)	.017	0.055
	No change	39 (59.0)	46 (43.8)		
	Improved	7 (10.6)	5 (4.8)		
Due to the pandemic, ability to meet basic needs (including housing, food, essential supplies, etc.)	Worse	12 (18.1)	52 (49.5)	<.001	0.005
	No change	48 (72.7)	50 (47.6)		
	Improved	6 (9.1)	3 (2.9)		
Due to the pandemic, life overall	Negative	47 (72.3)	101 (97.1)	<.001	0.005
	No impact	15 (23.1)	1 (1.0)		
	Positive	3 (4.6)	2 (1.9)		

* Based on responses from 169 people in this sample who indicated being currently treated with opioid medications.

† False Discovery Rate (*P* value corrected for multiple comparisons).

The subsequent comparison of parent-study collected demographic and clinical characteristics (Table 5) indicated an overall worse health profile among those in Class 2 compared to Class 1 participants, with Class 2 showing significantly (*P* < .05) worse pain interference, mental health-related quality of life, negative affect, opioid misuse risk, and pain catastrophizing. Compared to Class 1, Class 2 participants also tended to be younger, female, lonelier, less loving of oneself, viewing their back pain as terrible and not going to get better, and more frequently reporting lost house/school work days. No statistically significant differences were observed between the Classes in lost out-of-house work days (among the 35 “working now” participants), or in healthcare utilization reports.

**Table 5:** Comparison of demographic characteristics health-related outcomes between Class 1 and Class 2 participants.

Variable	Total (n = 172)	Class 1 (n = 66)	Class 2 (n = 106)	*P* value	FDR*
Demographics					
Gender, n (%)					
Female	108 (62.7)	35 (53.0)	73 (68.8)	.036	0.094
Male	64 (37.2)	31 (46.9)	33 (31.1)		
Age, years, mean (SD)	58.7 (10.6)	61.5 (11.0)	57.0 (10.1)	.009	0.036
Ethnicity, n (%)					
Hispanic or Latino	8 (4.8)	2 (3.2)	6 (5.8)	.451	0.0.601
Not Hispanic or Latino	157 (95.1)	60 (96.8)	97 (94.2)		
Race, n (%)					
Black/African American	9 (5.4)	3 (4.7)	6 (5.8)	.392	0.565
White	150 (90.4)	59 (93.6)	91 (88.3)		
Other	7 (4.2)	1 (1.5)	6 (5.8)		
Education, n (%)					
No high school diploma	3 (1.7)	2 (3.0)	1 (.94)	.743	0.787
High school graduate or GED	19 (11.0)	8 (12.1)	11 (10.4)		
Some college, no degree	34 (19.7)	14 (21.2)	20 (18.8)		
Occupational/technical/vocational program	9 (5.2)	2 (3.0)	7 (6.6)		
Associate degree: academic program	27 (15.7)	7 (10.6)	20 (18.8)		
Bachelor’s degree	41 (23.8)	16 (24.2)	25 (23.5)		
Master’s degree	29 (16.8)	13 (19.7)	16 (15.1)		
Professional school degree	4 (2.3)	1 (1.5)	3 (2.8)		
Doctoral degree	6 (3.5)	3 (4.5)	3 (2.8)		
Employment, n (%)					
Working now	32 (18.6)	11 (16.6)	21 (19.8)	.748	0.787
Unemployed	5 (2.9)	2 (3.0)	3 (2.8)		
Disabled	27 (15.57)	9 (13.6)	18 (16.9)		
Disabled due to back pain	61 (35.4)	22 (33.3)	39 (36.7)		
Retired	36 (20.9)	18 (27.2)	18 (16.9)		
Other (includes Other, Sick or maternity leave, Student, Keeping house)	11 (6.4)	4 (6.0)	7 (6.6)		
Back pain history and perception characteristics (NIH MD)					
CLBP duration, n (%)					
3–6 months	1 (0.6)	0 (0.0)	1 (.94)	.717	0.787
6 months–1 year	4 (2.3)	2 (3.0)	2 (1.8)		
1–5 years	25 (14.5)	8 (12.1)	17 (16.0)		
>5 years	142 (82.6)	56 (84.5)	86 (81.1)		
Prior treatments for back pain, Yes, n (%)					
Injections *(e.g., epidural steroid, facet*)	140 (81.4)	50 (75.7)	90 (84.9)	.188	0.358
Exercise therapy (physical therapy)	160 (93.0)	61 (92.4)	99 (93.4)	.402	0.565
Psychological counseling	62 (36.0)	24 (36.4)	38 (35.8)	.966	0.966
History of a low back operation, n (%)					
Yes	51 (59.3)	20 (54.0)	31 (63.2)	.682	0.787
One operation	32 (18.6)	16 (24.2)	16 (15.1)		
More than one operation	54 (31.4)	21 (31.8)	33 (31.1)	.376	0.563
No	85 (49.4)	29 (43.9)	56 (52.8)		
I feel that my back pain is terrible and it’s never going to get any better…, n (%)					
Agree	82 (47.6)	28 (42.4)	54 (50.9)	.038	0.094
Disagree	81 (47.1)	31 (46.9)	50 (47.2)		
It’s not really safe for a person with my back problem to be physically active…, n (%)					
Agree	60 (34.8)	21 (31.8)	39 (36.7)	.654	0.787
Disagree	108 (62.7)	44 (66.6)	64 (60.4)		
Clinical characteristics					
Average pain severity (BPI, 0–10 score), mean (SD)	5.9 (1.5)	5.7 (1.6)	6.0 (1.4)	.126	0.252
Function					
Pain interference (BPI, 0–10 score range), mean (SD)	6.4 (1.9)	5.9 (2.0)	6.7 (1.7)	.012	0.042
Back pain-related disability (ODI, 0–100 score range), mean (SD)	47.4 (13.6)	46.1 (14.5)	49.5 (12.9)	.109	0.236
Health-related quality of life (SF-12)					
Physical health composite score (0–100 range), mean (SD)	28.2 (8.6)	27.9 (9.2)	28.3 (8.3)	.723	0.787
Mental health composite score (0–100 range), mean (SD)	43.7 (11.4)	48.1 (10.5)	40.9 (11.1)	<.001	0.005
Daily dose of prescribed opioids, past 14 days (TLFB, morphine-equivalent mg/day), average daily dose, mean (SD)	196.2 (786.1)	227.7 (819.3)	176.6 (768.1)	.679	0.787
Opioid misuse risk (COMM), total score (0-35 range), mean (SD)	8.9 (6.2)	6.8 (4.6)	10.3 (6.6)	<.001	0.005
Sleep quality (NIH MD), total score (4-19 range), mean (SD)	10.1 (3.8)	10.6 (3.7)	9.8 (3.8)	.193	0.358
Negative affect (HADS), total score (1-36 range), mean (SD)	16.2 (7.7)	13.7 (7.8)	17.7 (7.2)	<.001	0.005
Pain catastrophizing (PCS), total score (0-50 range), mean (SD)	19.4 (11.5)	16.6 (10.5)	21.2 (11.8)	.010	0.037
Loneliness (UCLA LS), total score (3-9 range), mean (SD)	5.7 (2.1)	5.3 (2.2)	6.0 (2.1)	.034	0.093
Feeling loved (FLI)					
Feeling loved by others (0-100 score range), mean (SD)	80.1 (21.7)	82.0 (21.0)	78.9 (22.1)	.362	0.563
Loving yourself (0-100 score range), mean (SD)	69.7 (27.8)	75.1 (23.6)	66.3 (29.8)	.049	0.116
Healthcare utilization and productivity characteristics (past 6 months)					
Hospitalized, Yes, # (%)	27 (15.8)	6 (9.2)	21 (19.8)	.118	0.245
Number of nights hospitalized (n = 27), mean (SD)	10.5 (18.7)	8.8 (14.0)	11.0 (20.1)	.808	0.824
ED visit, Yes, # (%)	47 (27.5)	14 (21.5)	33 (31.1)	.252	0.437
Number of ED visits (n = 47), mean (SD)	2.5 (3.1)	1.6 (0.9)	3.0 (3.6)	.218	0.391
Clinic visit (“regular” care, urgent care), Yes, # (%)	148 (87.5)	55 (84.4)	93 (89.4)	.336	0.549
Number of clinic visits (n = 148), mean (SD)	7.6 (9.6)	6.6 (6.0)	8.0 (11.1)	.379	0.563
Mental health care visit, Yes, # (%)	61 (35.9)	19 (27.7)	42 (40.4)	.093	0.210
Number of mental health care visits (n = 61), mean (SD)	9.7 (8.5)	10.8 (10.3)	8.9 (7.5)	.434	0.594
Addiction care visit, Yes, # (%)	6 (3.5)	3 (4.6)	3 (2.8)	.546	0.693
Number of addiction care visits (n = 6), number, mean (SD)	11.8 (20.3)	3.0 (2.6)	20.7 (28.0)	.338	0.549
Whole workdays missed (among those “working now”), Yes, # (%)	35 (27.3)	12 (18.5)	23 (21.7)	.469	0.610
Number of whole workdays missed (n = 35), mean (SD)	47.2 (63.4)	54.7 (66.7)	43.3 (62.7)	.623	0.771
Chores (school/house work) missed, Yes, # (%)	121 (78.5)	41 (61.5)	80 (75.5)	.026	0.075
Number of whole days of chores missed (n = 121), mean (SD)	45.1 (49.8)	44.7 (56.3)	44.5 (46.6)	.757	0.787

BPI = Brief Pain Inventory, CLBP = Chronic Low Back Pain, COMM = Current Opioid Misuse Measure, ED = Emergency Department, FDR = False Discovery Rate, FLI = Feeling Loved Instrument, HADS = Hospital Anxiety Depression Scale, HCUPS = HealthCare Utilization and Productivity Survey, NIH MD = NIH Minimal Data Set, PCS = Pain Catastrophizing Scale, SF-12 = 12-Item Short Form Health Survey, TLFB = Timeline Followback, UCLA LS = UCLA Loneliness Scale.

* False Discovery Rate (*P* value corrected for multiple comparisons).

The False Discovery Rate correction for multiple comparisons (detailed in the Analytical Approach) showed maintained statistical significance for the majority of the above comparisons.

## 4. Discussion

This survey study documented the dramatic toll of the COVID-19 pandemic experienced by adults with advanced, refractory CLBP who had been treated with long-term opioids (N = 480). According to the survey responses, the pandemic affected all domains of relevant health-related outcomes as well as healthcare access, general wellbeing, and financial stability. The survey also indicated an exemplary adherence to public health recommendations aimed at preventing the COVID-19 infection, a finding that aligned with a low rate of self-reported COVID-19 infections and hospitalizations. Although this population as a whole was substantially impacted, a more nuanced evaluation, using a latent class analysis (n = 172), revealed statistically significantly differences in the pandemic-related harms between 2 identified classes of respondents; Class 2 (n = 106) reported uniformly worse pandemic impact than Class 1 (n = 66), along with an overall compromised profile of health- and quality of life-related measures. These findings highlighted the heterogeneity of experiences among adults with opioid-treated CLBP.

The vast majority of the survey respondents reported worsened pain, function, mental health and quality of life due to the pandemic. Under optimal circumstances, worse medical and mental health status would have led to increased intensity of clinical care to stabilize and improve such exacerbations, but in this mid-pandemic sample, nearly half of the respondents reported worsened access to healthcare, with approximately 10% reporting worse access to their opioid medications, and close to 20% also noting an increased use of opioid medications. In a time of pandemic crisis, with worsening health status, vulnerable populations such as those with opioid-treated CLBP should be able to maintain or increase access to healthcare services, but this did not happen.^[29]^

Although it might be tempting to apply unified “labels” to this population (i.e. this at-risk population experienced pandemic-related harms), the latent class analysis demonstrated significant heterogeneity, and that those who were affected most across one domain were also negatively impacted across other domains. Because of challenges across numerous domains of mental, medical, and socioeconomic factors, we concluded that a comprehensive evaluation and multi-modal approach would be needed to address the multiple issues detrimental to health, quality of life, and well-being. At the same time, this analysis underscored the need for an individually-tailored care plan – while some individuals may opt for addressing several issues at once, others may prefer to focus on a single problem. Participants’ qualitative comments offered a window into unique experiences, further emphasizing the need for personalized approach. Stakeholder feedback obtained as a part of this study corroborated these assumptions by making it clear that a “cookie-cutter” strategy is neither effective nor welcomed by patients.

For participants who were particularly affected by the pandemic (Class 2 in the latent class analysis), the findings of somewhat lower daily opioid dose (without statistically significant differences between the Classes), along with increased opioid misuse risk score (COMM questionnaire) are somewhat puzzling. Survey data cannot be used to infer causality, and can be interpreted in several ways. For example, we could hypothesize that opioid tapering or discontinuation, with subsequent lower daily opioid dose, may have been initiated by providers due to pandemic-related concerns, potentially resulting in elevated COMM scores (suggesting opioid misuse or opioid use disorder). It is also possible that those subjected to opioid tapers may have needed to resort to aberrant opioid use to cope with taper-related negative consequences (e.g., opioid withdrawal, worsened pain). The reports of pandemic-caused changes in opioid dose, and worse medication and general healthcare access particularly among Class 2 participants suggest that the latter could be the case. The qualitative data corroborated this notion that pandemic-related restrictions and care disruptions were common and impacted both opioid therapy and other chronic pain-specific management, with negative consequences for physical and mental health, and general well-being. Future prospective studies focused on these issues will be needed to better understand these associations and pathways.

Our results support recent findings regarding perceived pain experiences during the COVID-19 pandemic. For example, Chatkoff et al^[39]^ surveyed 487 patients with musculoskeletal, neuropathic, or postsurgical pain, and found increased treatment delays, worsened negative affect and perceived decline in treatment quality, and greater pain, with a third of respondents reporting more pain medication use when compared to pandemic’s onset. Similarly, a survey of 140 patients with opioid-treated non-cancer pain by Scherrer et al^[40]^ reported increased pain and pain interference during the first months of the COVID-19 pandemic. However, only 4% of their respondents reported increased use of prescription opioids due to COVID-19, lower than our reported rate. In that study, the authors hypothesized that the large proportion of participants using lower-dose opioids in an intermittent way may have contributed to their findings. In contrast, Amelot et al^[41]^ reported benefits attributed to the pandemic among 55 adults with CLBP, with 30% of them treated with opioids, one-month after a COVID-19-related lockdown in France, compared to pre-pandemic. The authors reported that the emotional distress related to CLBP had paradoxically improved by the pandemic-generated stress and anxiety, hypothesizing the perceived life-threatening stress of COVID-19 may have eclipsed or altered the painful perceptions of CLBP. The majority of our significantly larger sample did not report favorable experiences due to the pandemic; in fact, the opposite was true, with many noting worsened outcomes through both quantitative and qualitative responses.

Perhaps most intriguing, a longitudinal study by Mun et al^[42]^ showed, using repeated assessments over time, that the deleterious pandemic-related effects had not worsened as the pandemic continued evolving. In this study, individuals with chronic pain did not report significant progressive exacerbation of pain, emotional distress, or opioid misuse risk when measured at 3 different timepoints from April 2020 to May 2021.^[42]^ Similar to our findings, that study reported heterogeneity in pain experiences, with socioeconomically-driven disparities noted in pain and depressive symptoms. It is worth noting that a substantial portion of that sample (44%) did not complete their follow-up, with younger, male, Hispanic participants and those with a lower educational level and more severe symptoms at baseline being more likely to miss their one-year assessments.^[42]^

Our study cohort reported increased loneliness during the pandemic, an unsurprising finding which aligns with other studies, and may contribute to poor health outcomes. One meta-analytic review found loneliness to be associated with an approximately 30% higher risk of premature mortality, an effect comparable to smoking, diabetes, obesity and other well-established risk factors.^[43]^ In our sample (N = 480), close to 60% of participants screened positive for loneliness, exceeding the rate among those living with paraplegia or tetraplegia after a spinal cord injury (with aproximately one-fourth of 175 participants screening positive) ^[44]^ Qualitative descriptions by our participants highlighted the devastating impact of loneliness and isolation caused by the pandemic, and were often heart-breaking.

Women in our study were more likely than men to belong to the LCA-identified Class 2 of responses, which indicated more severe pandemic-related harms. This is consistent with literature documenting worse impact of opioid-treated chronic pain among women pre-pandemic,^[45]^ and worse pandemic-related psychological distress.^[46,47]^ This could be due to many factors, including increasing burdens of childcare, homeschooling, and household responsibilities, often juggled with professional duties. Those in Class 2 showed significantly higher pain catastrophizing scores and negative affect scores that might have accounted, to some degree, for the higher COMM scores and the greater self-reported problems coping during the COVID-19 pandemic.

There are several limitations to note when interpreting the results of this cross-sectional survey. First, we relied on self-report, which inherently involves recall deficiencies and related biases; unfortunately, we have no pre-pandemic baseline data to compare. Also, we did not have objective ways to corroborate aberrant opioid use behaviors or the recommended behaviors to reduce the spread of COVID. Additionally, the self-selected respondents were mostly white, non-Hispanic/Latino, with at least some college-level education, and were recruited from participants in the larger, parent RCT, limiting generalizability to other populations. Nevertheless, we feel it is likely the responses from a more diverse sample would have pictured even higher pandemic-related burden. It is also possible that those most versus least severely impacted by the pandemic were less likely to respond to the survey, introducing potential bias. We did not determine whether barriers to telehealth (e.g., lack of computer expertise) contributed to loss of contact or reduced comprehensive care interventions. Despite these limitations, the results of this survey suggest that certain individuals with opioid-treated CLBP are prone to higher risk for increased isolation, mood disorder, greater disability, and potential opioid misuse during a pandemic, which disrupted usual healthcare. Identification of those who are most at risk and developing multimodal strategies to help mitigate the risks will be needed if we wish to help reduce the deleterious effects of future pandemics.

## 5. Conclusions

The pandemic substantially affected all domains of relevant health-related outcomes as well as healthcare access, general wellbeing, and financial stability among adults with opioid-treated CLBP. A more nuanced evaluation revealed a heterogeneity of experiences, underscoring the need for both increased overall support for this population and for an individualized approach to mitigate harms induced by pandemic or similar crises.

## Acknowledgments

*The reported work was funded through a Patient-Centered Outcomes Research Institute (PCORI) Award* (OPD-1601-33860). *The views and statements in this publication are solely the responsibility of the authors and do not necessarily represent the views of the PCORI, its Board of Governors or Methodology Committee.* This project also received funding and institutional support through University of Wisconsin-Madison School of Medicine and Public Health, and its Department of Family Medicine and Community Health; Brigham and Women’s Hospital, Harvard Medical School; University of Utah College of Social Work; and Pennsylvania State University Department of Family and Community Medicine.

We would like to thank our STAMP Study research team members for their help in collecting the study data. We would also like to acknowledge our Patient, Family, and other Stakeholder Advisors for their input on the study design, conduct, and result interpretation (Chantelle Thomas, Cheryl Wittke, Evan Nelson, Elizabeth Jacobs, Penney Cowan, Christin Veasley, Vicki Goodman-Strenski, Donald Folberg, Randy Coloni, Anna Goodman-Strenski, Lori Hansen, Alice Morehouse, David Morehouse, Michael Olson, Andy Olson).

## Author contributions

**Conceptualization:** Aleksandra E. Zgierska, Cindy A. Burzinski, Eric L. Garland, Bruce Barrett, Robert P. Lennon, Roger L. Brown, Yoshio Nakamura, Robert N. Jamison, Robert R. Edwards.

**Data curation:** Aleksandra E. Zgierska, Cindy A. Burzinski, Eric L. Garland, Bruce Barrett, Roger L. Brown, Anthony R. Schiefelbein, Yoshio Nakamura, Robert N. Jamison, Robert R. Edwards.

**Formal analysis:** Aleksandra E. Zgierska, Eric L. Garland, Bruce Barrett, Robert P. Lennon, Roger L. Brown, Anthony R. Schiefelbein, Yoshio Nakamura, Robert N. Jamison, Robert R. Edwards.

**Funding acquisition:** Aleksandra E. Zgierska, Eric L. Garland, Bruce Barrett, Robert N. Jamison, Robert R. Edwards.

**Investigation:** Aleksandra E. Zgierska, Cindy A. Burzinski, Eric L. Garland, Bruce Barrett, Robert P. Lennon, Yoshio Nakamura, Robert N. Jamison, Robert R. Edwards.

**Methodology:** Aleksandra E. Zgierska, Cindy A. Burzinski, Eric L. Garland, Bruce Barrett, Robert P. Lennon, Roger L. Brown, Anthony R. Schiefelbein, Yoshio Nakamura, Barbara Stahlman, Robert N. Jamison, Robert R. Edwards.

**Project administration:** Aleksandra E. Zgierska, Cindy A. Burzinski, Eric L. Garland, Bruce Barrett, Yoshio Nakamura, Robert N. Jamison, Robert R. Edwards.

**Resources:** Aleksandra E. Zgierska, Eric L. Garland, Bruce Barrett, Robert R. Edwards.

**Supervision:** Aleksandra E. Zgierska, Eric L. Garland, Bruce Barrett, Roger L. Brown, Yoshio Nakamura, Robert N. Jamison, Robert R. Edwards.

**Writing – original draft:** Aleksandra E. Zgierska, Eric L. Garland, Bruce Barrett, Robert P. Lennon, Roger L. Brown, Barbara Stahlman, Robert R. Edwards.

**Writing – review & editing:** Aleksandra E. Zgierska, Cindy A. Burzinski, Eric L. Garland, Bruce Barrett, Robert P. Lennon, Roger L. Brown, Anthony R. Schiefelbein, Yoshio Nakamura, Barbara Stahlman, Robert N. Jamison, Robert R. Edwards.

## Supplementary Material
