# Supplementary material for: Experiences of adults with opioid-treated chronic low back pain during the COVID-19 pandemic: A cross-sectional survey study
Source: Medicine (Baltimore). 2023 Oct 13;102(41):e34885. doi: 10.1097/MD.0000000000034885 (PMC10578753; doi:10.1097/MD.0000000000034885)
Supplement: Supplementary file 1 [file medi-102-e34885-s001.docx]

**Opioid-treated Chronic Low Back Pain: COVID-19 Impact Survey**

*INSTRUCTIONS: Please answer the following questions related to* ***how the COVID-19 pandemic has impacted*** *your life, chronic pain experiences, and access to resources. Please read each question carefully and select the response that is most true for you.*

1. What response best describes how **the COVID-19 pandemic** has impacted your pain on average in the past month? In the past month, my pain on average has:
   1. Very much increased
   2. Much increased
   3. Minimally increased
   4. Not changed
   5. Minimally decreased
   6. Much decreased
   7. Very much decreased
2. What response best describes how, during the past month, **the COVID-19 pandemic** has impacted your general activity? In the past month, my general activity has been:
   1. Very much worse
   2. Much worse
   3. Minimally worse
   4. Not changed
   5. Minimally improved
   6. Much improved
   7. Very much improved
3. What response best describes how, in the past month, **the COVID-19 pandemic** has impacted your enjoyment of life? In the past month, my enjoyment of life has been:
   1. Very much worse
   2. Much worse
   3. Minimally worse
   4. Not changed
   5. Minimally improved
   6. Much improved
   7. Very much improved
4. What response best describes how, in the past month, **the COVID-19 pandemic** has impacted your ability to cope with pain? In the past month, my ability to cope with pain has been:
5. Very much worse
6. Much worse
7. Minimally worse
8. Not changed
9. Minimally improved
10. Much improved
11. Very much improved
12. What response best describes how, in the past month, **the COVID-19 pandemic** has impacted your mental health which can include stress, anxiety or depression symptoms? In the past month, my mental health has been:
13. Very much worse
14. Much worse
15. Minimally worse
16. Not changed
17. Minimally improved
18. Much improved
19. Very much improved
20. What response best describes how **the COVID-19 pandemic** has impacted your access to healthcare (including to your regular healthcare provider and specialty treatments)? My access to healthcare has been:
21. Very much worse
22. Much worse
23. Minimally worse
24. Not changed
25. Minimally improved
26. Much improved
27. Very much improved
28. Are you currently being treated with any type of opioid medication?

Yes No

7a) What response best describes how, in the past month, **the COVID-19 pandemic** has impacted your **use** of prescribed opioid medications? In the past month, my **use** of prescribed opioid medications has been:

- 1. Very much increased
  2. Much increased
  3. Minimally increased
  4. Not changed
  5. Minimally decreased
  6. Much decreased
  7. Very much decreased

7b) What response best describes how, in the past month, **the COVID-19 pandemic** has impacted your **access** to prescribed opioid medication? In the past month, my **access** to opioid medications has been:

1. Very much decreased
2. Much decreased
3. Minimally decreased
4. Not changed
5. Minimally increased
6. Much increased
7. Very much increased
8. What response best describes how **the COVID-19 pandemic** has impacted your financial situation (including income, savings, ability to pay bills, etc.)? My financial situation has been:
9. Very much worse
10. Much worse
11. Minimally worse
12. Not changed
13. Minimally improved
14. Much improved
15. Very much improved
16. What response best describes how **the COVID-19 pandemic** has impacted your ability to meet your basic needs (including housing, food, essential supplies, etc.)? My ability to meet my basic needs has been:
17. Very much worse
18. Much worse
19. Minimally worse
20. No change
21. Minimally improved
22. Much improved
23. Very much improved
24. What response best describes your personal experience with **the COVID-19 virus infection**?
    1. I was diagnosed with COVID-19, and this diagnosis was confirmed by a laboratory test.
    2. I was suspected to have COVID-19 infection, but have not been tested or this diagnosis has not been confirmed by a laboratory test.
    3. Other (please specify): ________________________________________

10a. Were you hospitalized (or did you have to stay in the hospital overnight) due to the COVID-19 infection?

Yes No

If yes, how many nights did you spend at the hospital due to the COVID-19 infection?

# nights ____________

1. What response best describes how the **COVID-19 pandemic** has impacted your life overall?
2. Very negatively overall
3. Somewhat negatively overall
4. A little bit negatively overall
5. Not impacted overall (negative and positive impacts equally balanced overall)
6. A little bit positively overall
7. Somewhat positively overall
8. Very positively overall
9. Please share with us how **the COVID-19 pandemic** has impacted your experience living with chronic back pain: _____________________________________________________
10. What else would you like to share with us about how the **COVID-19 pandemic** has impacted your life positively and/or negatively overall? _________________________________________
11. The next questions are about practicing public health recommendations to reduce the spread of COVID-19.

| Currently, how often do you… | Never | Rarely | Some of the time | Most of the time | All of the time | Not applicable |
| --- | --- | --- | --- | --- | --- | --- |
| a. …wash your hands often with soap and water for at least 20 seconds especially after you have been in a public place, or after blowing your nose, coughing, or sneezing? | ⚪ | ⚪ | ⚪ | ⚪ | ⚪ | ⚪ |
| b. …wear a cloth face cover (facemask) when out in public | ⚪ | ⚪ | ⚪ | ⚪ | ⚪ | ⚪ |
| c. …avoid touching your eyes, nose, and mouth with unwashed hands? | ⚪ | ⚪ | ⚪ | ⚪ | ⚪ | ⚪ |
| d. …cover your mouth and nose with a tissue when you cough or sneeze or use the inside of your elbow? | ⚪ | ⚪ | ⚪ | ⚪ | ⚪ | ⚪ |
| e. …stay at home when you feel unwell? | ⚪ | ⚪ | ⚪ | ⚪ | ⚪ | ⚪ |
| f. …stay at least 6 feet (about 2 arms’ length) from other people) when you are outside your home? | ⚪ | ⚪ | ⚪ | ⚪ | ⚪ | ⚪ |
| g. …stay out of crowded places and avoid mass gatherings? | ⚪ | ⚪ | ⚪ | ⚪ | ⚪ | ⚪ |
